# Improving Transmission Efficiency of Large Sequence Alignment/Map (SAM) Files

**DOI:** 10.1371/journal.pone.0028251

**Published:** 2011-12-02

**Authors:** Muhammad Nazmus Sakib, Jijun Tang, W. Jim Zheng, Chin-Tser Huang

**Affiliations:** 1 Department of Computer Science & Engineering, University of South Carolina, Columbia, South Carolina, United States of America; 2 Department of Biochemistry, Medical University of South Carolina, Charleston, South Carolina, United States of America; National Institutes of Health, United States of America

## Abstract

Research in bioinformatics primarily involves collection and analysis of a large volume of genomic data. Naturally, it demands efficient storage and transfer of this huge amount of data. In recent years, some research has been done to find efficient compression algorithms to reduce the size of various sequencing data. One way to improve the transmission time of large files is to apply a maximum lossless compression on them. In this paper, we present SAMZIP, a specialized encoding scheme, for sequence alignment data in SAM (Sequence Alignment/Map) format, which improves the compression ratio of existing compression tools available. In order to achieve this, we exploit the prior knowledge of the file format and specifications. Our experimental results show that our encoding scheme improves compression ratio, thereby reducing overall transmission time significantly.

## Introduction

One of the primary tasks in bioinformatics research is to collect and analyze large volume of genomic sequencing data. Modern sequencing instruments are generating at least hundreds of millions of short reads. Along with novel sequencing technologies, many new alignment tools have evolved that can perform efficient read mapping against large reference sequences. Alignments generated from these tools have different formats, which is not helpful for downstream processing. In order to simplify this process, a common alignment format, such as Sequence Alignment/Map (SAM) has been designed.

For the purpose of research, scientists and researchers need to transfer over the Internet large amount of alignment data represented in SAM format. Greater transmission delay has always been an obstacle in this process. Thus, reducing transmission delay has become imperative. One way to accomplish this goal is to apply a maximum lossless compression on the files to be transferred.

In this paper, we present SAMZIP, an encoding scheme specifically designed to work on SAM files, which improves compression ratio of the existing compression tools available. The goal is to achieve maximum compression ratio, thereby achieving minimum overall transmission time. We also demonstrate experimentally how our encoding scheme accomplishes this goal.

### The SAM format

**Figure 1:**
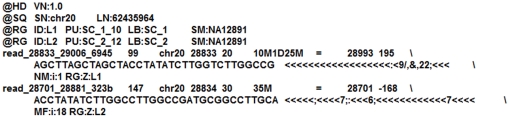
Example of a SAM file [**1**].

The SAM format comprises one header section and one alignment section. Both of the sections contain many lines delimited by the newline character. The fields inside a line are TAB delimited. The lines in the header section start with character ‘@’, while the lines in the alignment section do not. An example is shown in Figure 1. Each alignment line consists of 11 mandatory fields and a variable number of optional fields. A brief description of the mandatory fields is given in Table 1. The presence of these fields is mandatory although their value can be an asterisk (‘*’) or a zero if the corresponding information is unavailable. The optional fields are in the format: <TAG> : <VTYPE> : <VALUE>. Each tag is represented by two alphanumeric characters and appears only once for an alignment. A detailed description of each field has been given in the original SAM format specification [1].

**Table 1:** Mandatory Fields in the SAM format [1].

No.	Name	Description
1	QNAME	Query NAME of the read or the read pair
2	FLAG	Bitwise FLAG (pairing, strand, mate strand, etc.)
3	RNAME	Reference sequence NAME
4	POS	1-Based leftmost POSition of clipped alignment
5	MAPQ	MAPping Quality (Phred-scaled)
6	CIGAR	Extended CIGAR string (operations: MIDNSHP)
7	MRNM	Mate Reference NaMe (‘ = ’ if same as RNAME)
8	MPOS	1-Based leftmost Mate POSition
9	ISIZE	Inferred Insert SIZE
10	SEQ	Query SEQuence on the same strand as the reference
11	QUAL	Query QUALity (ASCII-33 = Phred base quality)

### The BAM format

The binary equivalent of SAM is called Binary Alignment/Map (BAM). It is compressed by the BGZF library; a generic library is developed by Li et al. [2]. It also provides fast random access in the compressed file.

### Encoding Techniques

The current compression technology includes a wide range of encoding techniques. We provide a background introduction for some of them, as they are needed for our own encoding scheme.

#### Run-length Encoding

One of the very basic encoding techniques is run-length encoding. Run-length encoding (RLE) is a technique in which a continuous occurrence of a data value is stored as a single data value and count, rather than as the original sequence. This technique is useful in compressing files that contain many such long runs of same data value.

#### Delta Encoding

Delta encoding is another technique where transmission of data takes place in the form of differences between successive values rather than the original values. This is also known as data differencing. This technique can be useful in encoding data values where there is a small difference value in consecutive data values. It is easier to encode the small difference values rather than encoding large data values.

#### Huffman Coding

Huffman coding is an entropy encoding algorithm developed by David A. Huffman in 1952. It is a popular algorithm for lossless data compression. The main idea is to construct a variable length code table for encoding each character in the source file. The variable-length code table is derived using the estimated probability of occurrence for each source character.

The resulting code is known as prefix-free code. It means that the bit string representation of a given symbol is never a prefix of the bit string representing any other symbol. It results into codes of shorter length for most common characters, while longer code lengths for less common characters. If there is a uniform probability distribution for the occurrence of the characters and the number of entries in the code table is a power of two, the Huffman coding becomes equivalent to simple binary encoding or ASCII encoding.

#### Dictionary Coding

A dictionary coding is a type of substitution coding where matched string is substituted by another string from the dictionary maintained by the encoder. Dictionaries can be one of two types - static and dynamic. In the static dictionary, the full set of strings is defined before coding begins and does not change during the coding process. This approach is particularly useful if the data to be encoded is fixed and large, and a pattern can be ascertained beforehand.

### Related Compression Research

In the recent years, researchers have been trying to come up with better compression tools to achieve efficient storage and transmission of genomic sequence data. Here, we will give a brief overview of some of the available compression tools developed over the past few years.

SLIMGENE is a domain specific lossless compression tool implemented by Kozanitis et al. [3] that can achieve 40× compression of genomic fragments without and 5× compression when the quality values are included. This tool mostly works with Illumina Export format which is one of the formats used for packaging and exporting genomic fragments. Another similar tool is DSRC (DNA Sequence Reads Compressor), developed by Deorowicz et al [4]. It works on genomic data in FASTQ format and has superior compression ratio over its competitor, G-SQZ. DRSC has a compression ratio of 4 to 6.5 over different variants of FASTQ format. Wang et al [5] presented GRS, a compression tool for storing and analyzing genome resequencing data. They tested it on the first Korean personal genome sequence data set, achieving about 159-fold compression.

## Materials and Methods

Our encoding scheme exploits two important characteristic of SAM files to improve compression ratio, and consequently the transmission time. First, the Alignment section of the SAM files consists of 11 mandatory fields and one optional field which may occur any number of times in one alignment record. All of these fields can be processed independently, since the fields have little inter-relation among them. Therefore, we can use parallel processing to process these fields and reduce processing time significantly. Secondly, we have a clear and specific format for each of these fields. We know the range of values that each of these fields may contain and also the approximation of occurrence of these values. We can utilize this knowledge to improve the encoding and achieve better compression ratio.

An important fact is that, all of the existing best case compression tools try to achieve the best compression ratio in one run. Hence, it is not possible to apply compression on an already compressed file. Even if this becomes possible, the second run does not improve compression ratio, rather it increases the output file size. Our goal is to encode the uncompressed file within least possible period of time and generate an intermediate encoded file with a moderate compression ratio, and then apply one of the best case compression tools on the intermediate file to achieve maximum compression ratio.

In our encoding algorithm, we used a combination of encoding techniques including run-length encoding, Huffman encoding, delta encoding, and dictionary coding. The usage of individual encoding techniques varied upon the pattern of data values we encountered in our dataset. We briefly describe the techniques for each of the fields as follows.

1. **QNAME:** This field exhibits a common long subsequence in the field values. If we remove this subsequence, the values become a set of numbers separated by delimiters. Our algorithm first calculates the longest common subsequence from these values and encodes it as a fixed value. Then, the remaining part is encoded as integer values (without the delimiters).
2. **FLAG:** This field contains small integer values fitting within a single byte or two, and exhibit frequent repetitions. We use run-length encoding here.
3. **RNAME:** This field mostly exhibits a few fixed values over the entire course of the file. Here, we use run-length encoding to encode these values which can include string types.
4. **POS:** This field contains large integer values which exhibit a constant increase and repetition. We combine run-length encoding and delta encoding for these values.
5. **MAPQ:** This field is encoded using the same technique applied for the FLAG fields.
6. **CIGAR:** This field contains values of alpha-numeric type of any length. They also exhibit long runs of the same value. We use run-length encoding here but include the length of the bytes for decoding purpose.
7. **MRNM:** This field can have any length of alpha-numeric values, but a careful observation of our dataset reveals that mostly this field contains either of the two values: ‘ = ’ and ‘*’. In this case, we use 1-bit encoding for these two values. In other cases, run-length encoding is used.
8. **MPOS:** This field contains similar values as the POS field, but without the constant increase in the values. We use the usual run-length encoding to encode this field, and include the byte length information for decoding purpose.
9. **ISIZE:** A constant value of 0 (zero) is found in most of the files for this field, which can be discarded totally and included in the dictionary of fixed values.
10. **SEQ:** This field normally contains a sequence of characters from the set {A, T, C, G}. These four characters can be encoded directly using only 2 bits. Some records may contain occasional ‘N’ characters, which is rare. We maintain the position of these ‘N’ characters and encode them separately. Currently, our algorithm is limited to support only ACGTN values for this field.
11. **QUAL:** This field has the characteristic of long runs of single character value, which is suitable for using run-length encoding.
12. **TAG:** This is an optional field, having the format: <TAG> : <VTYPE> : <VALUE>. The TAG and VTYPE portions can have two and one character values respectively, taken from a limited set of values. So, these fields are particularly suitable for Huffman encoding. The VALUE portion contains variable length alphanumeric values, which are kept as it is.

The encoded output is divided into separate files, one output file for each field. Any suitable compression tool can be applied on the encoded output files for further compression. The encoded files have a much smaller size compared to the original input file, so a large input file is no longer needed for the compression tools. This effectively reduces the compression time. After decompression, the decoding algorithm reads all of these files to reconstruct the original SAM file.

Our algorithm is based on assumptions on the values that the SAM fields should exhibit. If the input varies from the normal characteristic, the algorithm would exhibit worst performance. But it can be adjusted by changing different parameters for different fields. Another worst case scenario is when the input has absolutely zero repetitive values. On the other hand, this algorithm shows best case performance when there are long runs of repetitive occurrences in the data. As the algorithm is for lossless data compression, the data values are decoded exactly as they are encoded without any loss of information. We have tested the integrity of the data by matching the original file with the decompressed file.

In a SAM file, major portion of the data consists of QNAME, SEQ, and QUAL data values. QNAME values usually exhibit a pattern with a long subsequence of alphanumeric characters. This subsequence can be easily separated leaving only a few numeric values or symbols. So, the QNAME data can have high compression ratio depending on their pattern. SEQ values contain characters from a small fixed set and have high compression ratio. On the other hand, QUAL data values may contain characters from a large set and have high entropy. Hence, QUAL data have low compression ratio. The rest of the data values consist mostly of small integer values or strings of short length. Their compression ratio depends on the repetition of these data values.

## Results

We implemented SAMZIP in C in order to evaluate its performance. The source code of our SAMZIP implementation can be found in the supporting information file S1. A 10 MB sample input file for SAMZIP can be found in the supporting information file S2. We conducted several tests for both offline and online compression scenarios. For the offline compression scenario, we assume that data is already available and can be compressed ahead of time, so we only consider the effective transmission time (calculated based on the reduced data size). For the online compression scenario, we assume that data needs to be compressed on the fly, so the compression time is also added to the effective transmission time to get the total transmission time. We compared our scheme to two popular general purpose lossless compressors, gzip and WinRAR (both the normal compression mode and the best compression mode), and one SAM file specific compression tool, samtools. The test data comprised of five files from our existing dataset. The test machine was an Intel Xeon 2.8 GHz, Quad Core CPU, 6 GB RAM, under 64-bit Ubuntu Maverick Version 10.10 and Windows 7 Professional Edition environments.

Table 2 shows the experimental results for offline compression. For each of the compression tools, we show the *compressed file size* (in Gigabytes) and corresponding *transmission time* (in minutes). The transmission time is calculated based on the assumption of using T1 bandwidth (1.544 Mbps or 0.193 MBps). For example, an output file size of 3.5 GB can be transmitted in (3584/0.193) = 18569.94 seconds or 309.50 minutes. From Table 2, we can see that WinRAR has superior compression ratio over samtools and gzip, even in normal compression mode. If we compare samtools with WinRAR (best), the transmission time of WinRAR (best) improves roughly by approximately 115∼144 minutes. In the case of gzip, the improvement ranges from 76 to 98 minutes. Now, when we combine SAMZIP with WinRAR in the best compression mode, we get even better compression ratio and transmission time. The improvement in the transmission time of our method ranges from 6 to 21 minutes over the results of WinRAR (best).

**Table 2:** Offline Compression Results.

Original File Size [GB]	samtools		gzip		WinRAR(Normal)		WinRAR (Best)		SAMZIP +WinRAR(Best)	
	Comp.File Size[GB]	TransTime[min.]	Comp.File Size[GB]	Trans.Time[min.]	Comp.File Size[GB]	Trans.Time[min.]	Comp.File Size[GB]	Trans.Time[min.]	Comp.File Size[GB]	Trans.Time[min.]
23.13	3.5	309.50	3.06	270.59	2.89	255.56	2.2	194.54	2.08	183.93
25.01	3.6	318.34	3.09	273.24	2.9	256.44	2.19	193.66	2.08	183.93
25.25	3.74	330.72	3.22	284.74	3.04	268.82	2.28	201.62	2.21	195.43
25.39	4.29	379.36	3.77	333.37	3.51	310.38	2.66	235.22	2.42	214.00
25.81	3.92	346.64	3.4	300.66	3.19	282.09	2.41	213.11	2.24	198.08

Table 3 shows test results for online compression. In this table, only the total time is shown for each of the compression methods. The total time is calculated by adding the compression time, the effective transmission time, and the decompression time. For comparison purpose, we also list the result of WinRAR in the fastest compression mode, whose compression time is relatively short but compression ratio is inferior to the normal mode and the best mode. As before, we used the same assumption of T1 bandwidth for transmission. From Table 3, we can find again that WinRAR (best) has the best total time as a single compression tool. But if we use SAMZIP with WinRAR (normal), we get even better total time. This is because WinRAR (normal) mode has the lowest decompression time. The combination of this mode with SAMZIP beats even the WinRAR (best) mode.

**Table 3:** Online Compression Results.

Original File Size [GB]	samtools	gzip	WinRAR (Fastest)	SAMZIP +WinRAR (Fastest)	WinRAR (Normal)	SAMZIP +WinRAR (Normal)	WinRAR(Best)	SAMZIP +WinRAR (Best)
	Time[min.]	Time[min.]	Time[min.]	Time[min.]	Time [min.]	Time[min.]	Time[min.]	Time[min.]
23.13	354.70	346.75	333.79	291.29	286.87	266.05	276.67	272.76
25.01	365.08	350.82	340.76	299.28	289.64	272.69	280.61	278.41
25.25	380.61	364.72	355.76	311.04	302.38	285.28	292.86	296.82
25.39	429.47	414.77	407.10	348.83	346.70	315.37	333.38	325.91
25.81	397.21	385.96	371.43	325.35	319.09	296.38	309.65	303.45

In these test cases, we combined SAMZIP with WinRAR. However, similar tests can be performed using the combination of other available compression tools with SAMZIP, and we can show that SAMZIP improves performance of those tools.

## Discussion

Research in Bioinformatics largely depends on storage and manipulation of huge amount of data. Efficient transmission of these data is crucial for the advancement of research. In this paper, we have implemented SAMZIP, an encoding scheme for SAM files, which improves compression ratio of existing compression utilities available. Our experimental results show that it can achieve significant improvement in transmission time over open source and commercial compression tools. In order to accomplish this, we utilized the prior knowledge of the file format and specifications.

## Supporting Information

File S1 **SAMZIP source code.** The complete source code of SAMZIP implementation written in C.
(RAR)

File S2 **Sample SAM file.** A 10 MB sample input file for SAMZIP.
(SAM)
